# Thymidine kinase 1 concentration in pleural effusion is a diagnostic marker and survival predictor for malignant pleural effusion

**DOI:** 10.1002/jcla.22901

**Published:** 2019-04-15

**Authors:** Tian Tian, Jun Li, Wenjun Hu, Cuiling Sun, Jian Zhou

**Affiliations:** ^1^ Department of Medical Oncology Fuyang People's Hospital Fuyang China; ^2^ Department of Medical Oncology The First Affiliated Hospital of Anhui Medical University Hefei China

**Keywords:** diagnosis, malignant pleural effusion, prognosis, Thymidine kinase 1

## Abstract

**Objective:**

Thymidine kinase 1 (TK1) is a key enzyme in the pyrimidine salvage pathway. Increased TK1 concentration correlates with cell division. TK1 is an emerging biomarker in cancer diagnosis; however, its effectiveness in diagnosis and management for malignant pleural effusion (MPE) is unclear. We evaluated the diagnostic efficiency and prognostic value of pleural effusion TK1 (pTK1) concentration for MPE.

**Methods:**

From 2013 to 2017, 210 pleural effusion samples were collected from 160 patients diagnosed with MPE and 50 patients diagnosed with benign pleural effusion (BPE). TK1 concentrations in pleural effusion were measured by chemiluminescence dot blot assays. The median follow‐up was 12 months. We constructed a receiver‐operating characteristic (ROC) curve to find the optimal cutoff value for MPE diagnosis. The hazard ratios were estimated using a multivariable Cox proportional hazard model. A nomogram was drawn to illustrate the prognostic characteristics of MPE.

**Results:**

The TK1 concentration in pleural effusion was significantly higher in MPE than BPE (*P* < 0.001), and patients with MPE could be distinguished by an optimal cutoff value of 3.10 pmol/L with a sensitivity of 0.894 and a specificity of 0.800. The multivariate analysis suggested that pTK1 concentration was an independent predictor of survival in patients with MPE.

**Conclusions:**

The diagnostic and prognostic prediction of MPE may be improved by measuring pTK1 concentration and utilizing a multivariate nomogram.

## 1 INTRODUCTION

Malignant pleural effusion (MPE) is a common symptom experienced by advanced stage cancer patients. This condition affects 500‐700 individuals per million population and accounts for more than 125,000 hospital admissions every year in the United States.^1^, ^2^ The estimated median survival for patients with MPE is 3‐12 months.^1^ However, due to the development of local and systemic therapy, some recent studies have reported increased survival times.^3^, ^4^ Although there has been notable progress in explaining the pathophysiology of MPE, challenges remain in diagnosis and precise prognostic assessment.^5^, ^6^

Thymidine kinase 1 (TK1) is an emerging biomarker in cancer diagnosis and outcome prediction.^7^ TK1 is a key enzyme in the pyrimidine salvage pathway and plays an important role in DNA precursor synthesis.^8^ The TK1 concentration increase in proliferating cells and studies have demonstrated that elevated expression of TK1 is associated with tumor cell division and proliferation.^9^ Serum TK1 (sTK1) is considered a marker to diagnose malignancy in the early stage,^10^ and a previous analysis also showed that serum TK1 is an independent predictor of tumor recurrence and is a prognostic factor for several types of cancer.^11^

Biomarkers for the diagnosis and survival evaluation in pleural effusion have been extensively studied,^12^ and most soluble protein biomarkers are more effective when measured in pleural effusion than in serum.^13^ The use of pleural biomarkers offers a cost‐effective and minimally invasive method for MPE management.^14^

The primary aim of our study was to evaluate whether pTK1 can be used as a diagnostic biomarker for MPE. The secondary aim was to assess the prognostic value of pTK1 concentration in MPE. The third aim was to determine whether an effective nomogram could be created to predict MPE outcomes.

## 2 METHODS

### 2.1 Study population

A total of 210 patients who were diagnosed with pleural effusion for the first time and treated at the Fuyang People's Hospital (Anhui, China) were included in this study. The selection criteria were as follows: (a) for benign pleural effusion (BPE) patients, the effusion was reduced or disappeared after receiving antibiotic therapy, and a one‐year follow‐up showed no relapse and no sign of malignant disease. (b) For MPE patients, primary cancer was confirmed by pathological diagnosis, and malignant cells were found in pleural effusion by cytology. The exclusion criteria were patients under 18 years old or with an expected survival of less than 1 month. The patients were followed up for a median of 1 year. Informed consent was obtained from each patient. The ethics research council of our hospital approved the protocol of this study.

### 2.2 Clinical and biochemical measures

Performance status was assessed using the Eastern Cooperative Oncology Group Performance Status (ECOG PS) score. The pleural effusion samples were collected from the same pleural effusion sample sent for cytology examination. Each sample was stored at 4°C immediately after collection, and then, the TK1 concentration was measured in 24 h using a commercially available chemiluminescence dot blot assay kit (SSTK Biotech, Ltd., Shenzhen, China). Briefly, standards and effusion samples were directly transferred to a nitrocellulose membrane. Then, human anti‐TK1 chicken immunoglobulin Y antibody was added to the samples. Next, the light intensities of spots were captured by an imaging system (SSTK Ltd., Shenzhen, China). Finally, curves were created to calculate the concentration.

### 2.3 Statistical analysis

Normal distributed variables are shown as the mean and standard deviation. Categorical variables are expressed as absolute numbers with a percentage of subjects. Student's *t* test was used to compare continuous variables. Skewed distribution data are presented as medians with the 25th and 75th percentile, and a nonparametric test (Mann‐Whitney *U*) was conducted to evaluate differences in those data. Diagnostic accuracy was evaluated using a receiver‐operating characteristics (ROC) curve. Overall survival (OS) was defined as the date of MPE diagnosis to the date of death or last contact. The optimal diagnosis cutoff was identified as the point with the maximum value of Youden index (sensitivity + specificity‐1). Univariate analysis was performed for potential cofounders. Variables that were significantly associated with survival were selected for multivariate Cox regression analysis applying the backward stepwise method, and hazard ratios (HRs) were estimated using this model. A nomogram was created to illustrate the outcome of the prognostic factors on OS. The concordance index (C‐index) was calculated to assess the discrimination ability of this nomogram. Internal validation was conducted by 1000 bootstrap resamples to obtain an unbiased estimate of model performance.

All analyses were conducted using SPSS software (version 25.0; IBM Corp., Armonk, NY, USA) and R software (version 3.5.2; www.r-project.org). The nomogram and the optimal cutoff values of continuous variables for survival analysis were determined by using R software with the survminer and rms packages. A *P* value < 0.05 was considered statistically significant.

## 3 RESULTS

### 3.1 Patient characteristics

In total, 210 patients who met the criteria were included in our study. The MPE group included 160 patients. The diseases leading to MPE were lung cancer (143 patients), breast cancer (five patients), esophageal cancer (five patients), gastric cancer (five patients), and mesothelioma (two patients). The BPE group consisted of 50 patients (parapneumonic effusion, 29 patients; tuberculous pleural effusion, 21 patients). Among the 160 patients with MPE, 73 were male and 87 were female, with a mean age of 59.7 ± 9.12. Regarding 50 patients with BPE, 23 were male and 27 were female, with a mean age of 57.3 ± 9.98.

### 3.2 Accuracy of pTK1 concentrations to diagnose MPE

**Figure 1:**
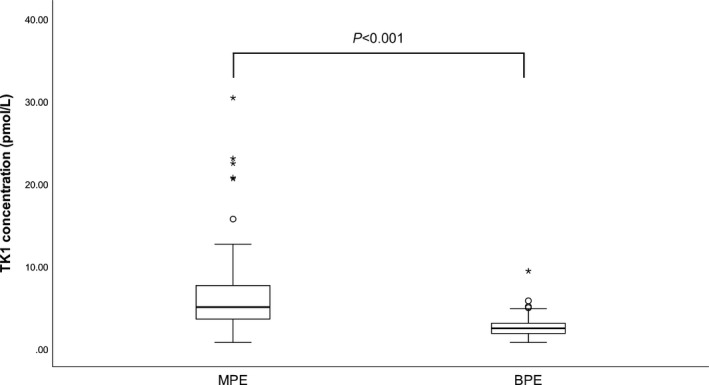
PTK1 concentrations categorized by different patient groups

**Figure 2:**
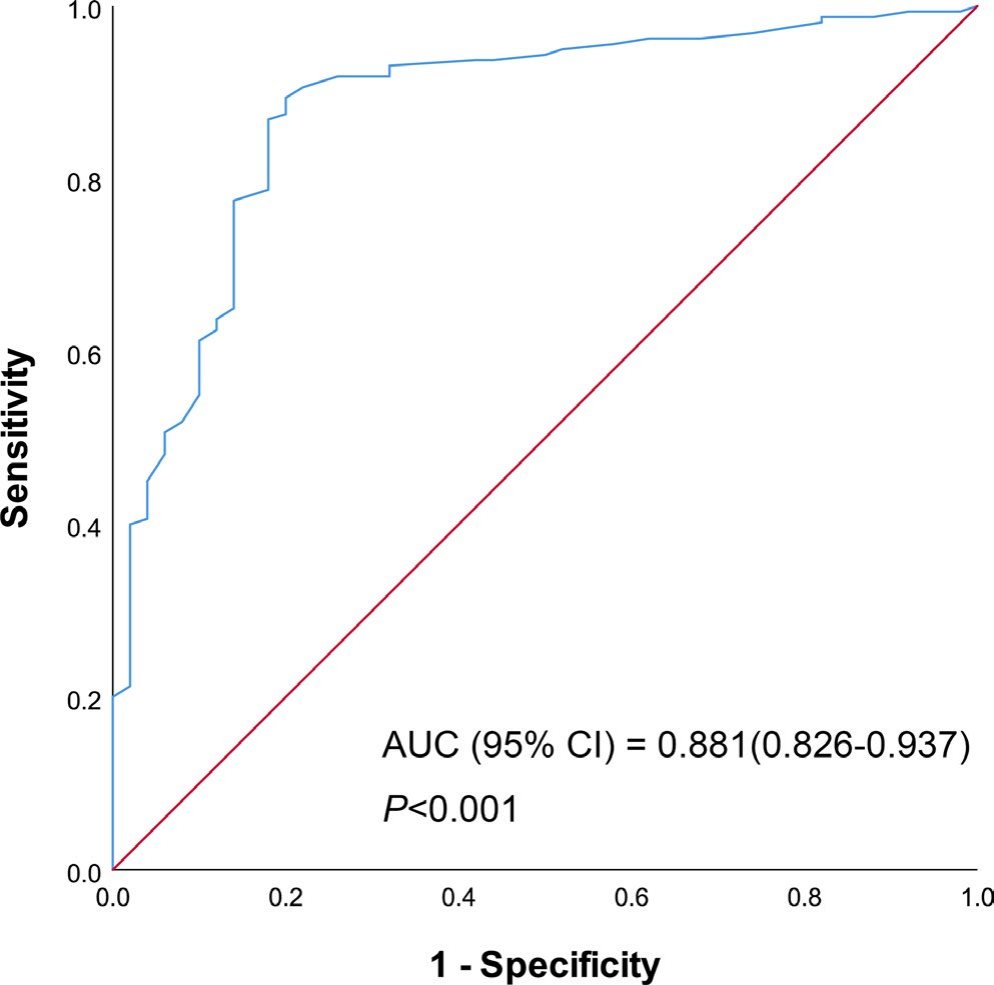
Receiver‐operating characteristic curve of pTK1 in the diagnosis of MPE

The concentration of pTK1 in cases of MPE was 5.01 (3.55‐7.63) pmol/L, while in cases of BPE, it was 2.44 (1.81‐3.05) pmol/L. The median value of pTK1 in MPE was significantly higher than that in BPE (*P* < 0.001, Figure 1. The ROC curve of pTK1 in MPE patients was plotted, and the AUC was 0.881 [95% confidence interval (CI): 0.826‐0.937, *P* < 0.001, Figure 2]. PTK1 revealed the highest sensitivity (0.894) and specificity (0.800), with an optimal cutoff value of 3.10 pmol/L.

### 3.3 PTK1 concentrations and prognosis

**Figure 3:**
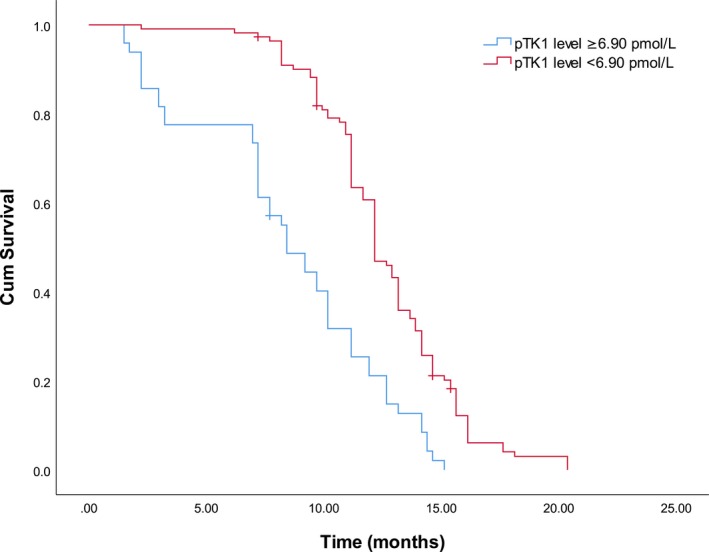
Kaplan‐Meier plot of patients with different pTK1 levels

Age, gender, smoking, ECOG PS, pathological type, smoking history, multiple metastasis, pTK1, sTK1, T stage, and N stage of primary cancer were regarded as potential cofounders, and Kaplan‐Meier univariate survival analysis was performed. Patients with elevated pTK1 had a significantly decreased OS. The median values for OS of patients with high pTK1 values and with low pTK1 values were 8.4 and 12.2 months, respectively Figure 3. Pathological type other than adenocarcinoma, multiple metastasis, and poor PS score were also associated with significantly decreased OS Table 1.

**Table 1:** Clinicopathological characteristics of patients with MPE and the associated median survival time

Characteristic	Cases, n (%)	median survival time	χ2	*P*
Age, years				
<60	77	335	0.544	0.461
≥60	83	365		
Gender				
Male	73	331	0.162	0.687
Female	87	351		
ECOG performance status score				
≤2	69	425	114.381	<0.001
>2	91	291		
Pathological type				
Adenocarcinoma	129	365	22.661	<0.001
Other pathological types	31	253		
Smoking history				
Yes	41	341	0.001	0.970
No	119	340		
Multiple metastasis				
Yes	42	335	21.894	<0.001
No	118	410		
Time interval between MPE and primary cancer diagnosis				
<60	66	321	4.706	0.030
≥60	94	354		
Serum TK1 level				
<2.00 pmol/L	71	356	0.817	0.366
≥2.00 pmol/L	89	329		
Pleural effusion TK1 level				
<6.90 pmol/L	111	365	36.111	<0.001
≥6.90 pmol/L	49	253		
N stage of primary cancer				
≤2	79	345	0.600	0.439
>2	81	336		
T stage of primary cancer				
≤2	80	350	0.064	0.800
>2	80	358		

### 3.4 Development and validation of the survival model

**Figure 4:**
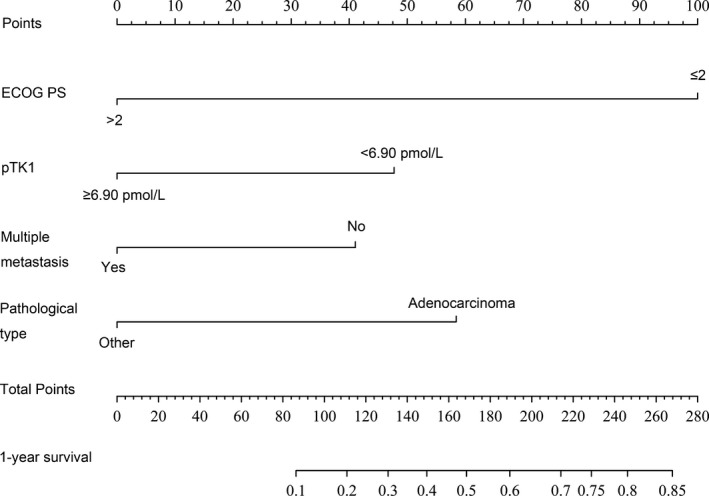
Nomogram for predicting 1‐year overall survival rates in patients with MPE

**Figure 5:**
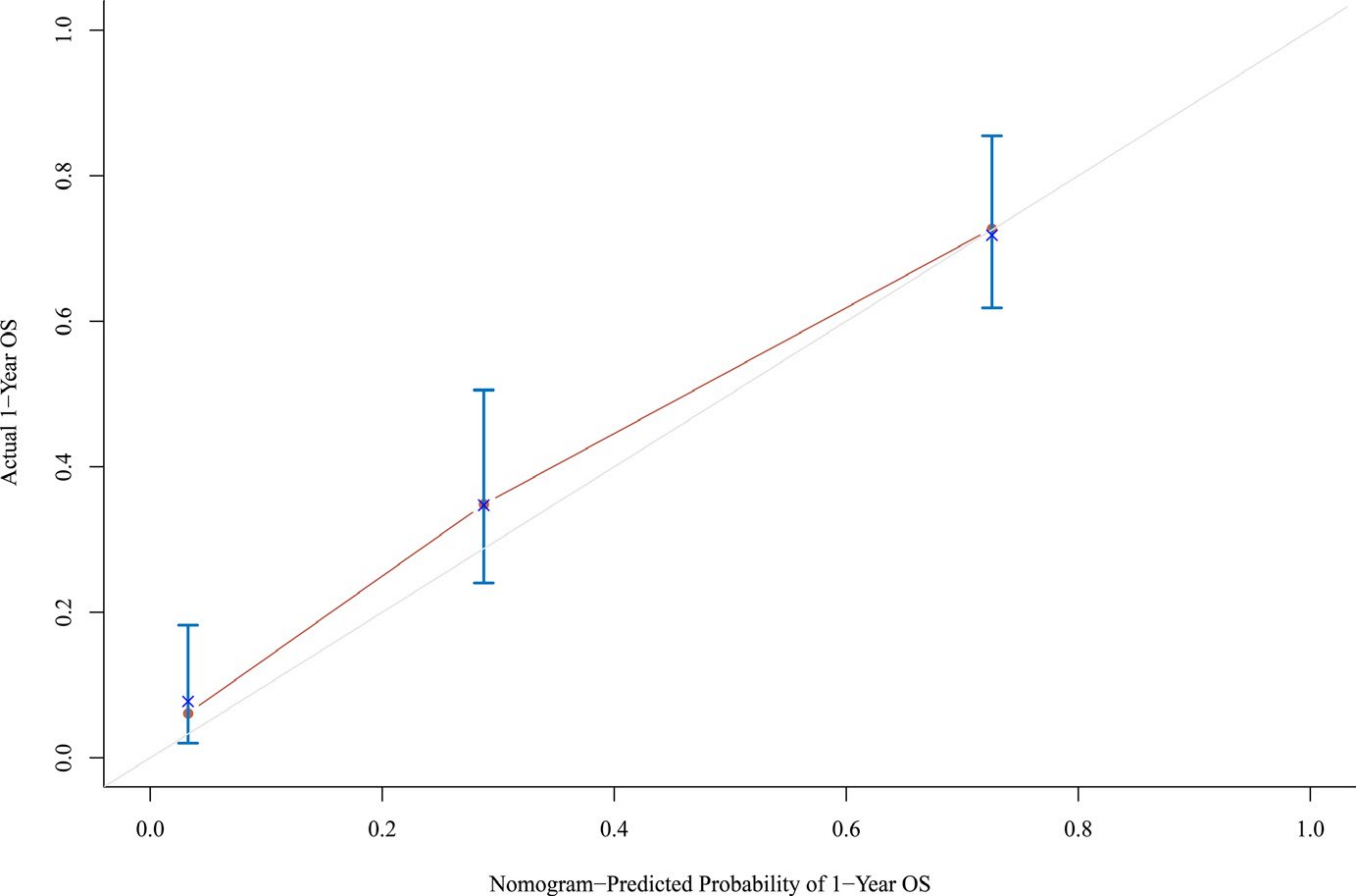
Calibration plot of the nomogram for the probability of 1‐year survival

Multivariate backward stepwise Cox regression analysis was conducted to identify independent factors from statistically significant variables (*P* < 0.05) proven by Kaplan‐Meier univariate analysis Table 2. This Cox model was then used to create a nomogram Figure 4. The C‐index for 1‐year OS prediction was up to 0.817 (95% CI: 0.792‐0.842), and the calibration plot for the probability of 1‐year survival exhibited an ideal agreement between nomogram‐predicted probability and actual survival Figure 5.

**Table 2:** Cox regression analysis of factors affecting patient prognosis

Characteristic	Regression coefficient	Standard error	Wald	HR	95% CI	*P*
Pathological type	−0.957	0.225	18.181	0.384	0.247‐0.596	<0.001
ECOG PS	−1.968	0.216	82.737	0.140	0.091‐0.214	<0.001
Pleural TK1 level	−0.871	0.196	19.857	0.418	0.285‐0.614	<0.001
Multiple metastasis	−0.700	0.208	11.343	0.496	0.330‐0.746	0.001

CI, confidence interval; HR, hazard ratio.

## 4 DISCUSSION

Although the gold standard of MPE diagnosis is cytopathology,^15^ occasionally, several sampling times were required for identification of malignant cancer cells with microscopy. Various markers have been suggested as noninvasive tests to help discriminate between BPE and MPE and to provide prognostic information.^16^, ^17^ The most heavily studied diagnostic biomarker in pleural effusion is carcinoembryonic antigen (CEA). A meta‐analysis including 49 studies suggested that the pooled sensitivity and specificity of pleural effusion CEA for diagnosing MPE were 0.549 and 0.962.^14^ In a previous study,^18^ the diagnostic value of TK, neuron‐specific enolase (NSE), CEA, and cytokeratin fragment 19 (CYFRA 21‐1) was determined, and the TK concentration was investigated using a radio enzyme assay. The TK concentration had the highest diagnostic accuracy (Youden index: 0.85) among the above markers. In our study, using a cutoff point of 3.10 pmol/L, the sensitivity and specificity of pTK1 for detecting MPE were 0.894 and 0.800, respectively. The LENT score system^19^ was calculated by four variables, including pleural fluid lactate dehydrogenase (LDH) level, ECOG PS, neutrophil‐to‐lymphocyte ratio (NLR), and tumor type, and it is widely used in risk stratification of MPE patients. However, a recent study suggested that it underestimates the prognosis in patients with MPE caused by lung adenocarcinoma.^20^ The recently published PROMISE score system^21^ includes a clinical score method and a biological score method, which is a clinical score plus tissue inhibitor of metalloproteinases 1 (TIMP1). However, this system can only predict the 3‐month mortality, and adding TIMP1 to the score system only contributed very modest effects, which limited its clinical value.^22^ In our study, pTK1 was proven to be significant in the univariate test, and multivariate Cox regression analysis demonstrated that pTK1 is an independent prognostic factor, contributing to a strong effect in the survival model.

TK exists in two forms: TK1 is found primarily in the cytoplasm, and TK2 is concentrated in mitochondria.^23^ Since TK1 is cell‐cycle regulated and TK2 is constitutively expressed, the value of sTK1 as a biomarker for diagnosis and its prognostic significance in lung cancer, breast cancer, esophageal cancer, and gastric cancer have been investigated in recent years,^24^, ^25^, ^26^ and our study analyzed the TK1 concentration in pleural effusion for the first time.

Nomograms have been created to assess survival factors in various malignancies.^27^ Nomograms are useful for visualizing prognostic factors and may help physicians make precise and individualized predictions of MPE outcomes. However, to the best of our knowledge, a nomogram to predict the probability of 1‐year survival for MPE patients has not been reported.

There are both strengths and limitations in our study. A major limitation of our study was that the sample size was relatively small and performed in a single institution. Another limitation is the lack of external validation for our survival model. Multicenter studies are required to validate the diagnostic and prognostic precision of this model.

## 5 CONCLUSION

We evaluated the value of measuring pTK1 concentration in the diagnostic and prognostic prediction of MPE and established a nomogram for predicting 1‐year survival. As pTK1 measurement is noninvasive and shows high diagnostic and prognostic value, it may be a useful biomarker in MPE management.

## CONFLICT OF INTEREST

The authors declare no conflict of interest.
